# Examining supporting and constraining factors of physicians’ acceptance of telemedical online consultations: a survey study

**DOI:** 10.1186/s12913-023-10032-6

**Published:** 2023-10-19

**Authors:** Sören Diel, Eileen Doctor, Riccardo Reith, Christoph Buck, Torsten Eymann

**Affiliations:** 1 Branch Business & Information Systems Engineering of the Fraunhofer FIT and FIM Research Center for Information Management, University of Bayreuth, Wittelsbacherring 10, 95444 Bayreuth, Germany; 2 Chair of General Business Management, University of Bayreuth, Universitätsstraße 30, 95447 Bayreuth, Germany; 3 Faculty of Informatics, Augsburg University of Applied Sciences and Branch Business & Information Systems Engineering of the Fraunhofer FIT, Alter Postweg 101, 86159 Augsburg, Germany; 4 QUT Business School, Centre for Future Enterprise, Queensland University of Technology, 2 George St, Brisbane, QLD-4000 Australia

**Keywords:** Telemedicine, Online consultations, Acceptance, UTAUT, Structural equation modeling

## Abstract

As healthcare demands exceed outpatient physicians’ capacities, telemedicine holds far-reaching potential for both physicians and patients. It is crucial to holistically analyze physicians’ acceptance of telemedical applications, such as online consultations. This study seeks to identify supporting and constraining factors that influence outpatient physicians’ acceptance of telemedicine.

We develop a model based on the unified theory of acceptance and use of technology (UTAUT). To empirically examine our research model, we conducted a survey among German physicians (*n* = 127) in 2018–2019. We used the partial least squares (PLS) modeling approach to test our model, including a mediation analysis.

The results indicate that performance expectancy (β = .397, *P* < .001), effort expectancy (β = .134, *P* = .03), and social influence (β = .337, *P* < .001) strongly impact the intention to conduct online consultations and explain 55% of its variance. Structural conditions regarding data security comprise a key antecedent, associating with performance expectancy (β = .193, *P* < .001) and effort expectancy (β = .295, *P* < .001). Regarding potential barriers to usage intentions, we find that IT anxiety predicts performance (β = –.342, *P* < .001) and effort expectancy (β = –.364, *P* < .001), while performance expectancy fully mediates (βdirect = .022, *P* = .71; βindirect = -.138, *P* < .001) the direct relationship between IT anxiety and the intention to use telemedical applications.

This research provides explanations for physicians’ behavioral intention to use online consultations, underlining UTAUT’s applicability in healthcare contexts. To boost acceptance, social influences, such as personal connections and networking are vital, as colleagues can serve as multipliers to reach convergence on online consultations among peers. To overcome physicians’ IT anxiety, training, demonstrations, knowledge sharing, and management incentives are recommended. Furthermore, regulations and standards to build trust in the compliance of online consultations with data protection guidelines need reinforcement from policymakers and hospital management alike.

**Supplementary Information:**

The online version contains supplementary material available at 10.1186/s12913-023-10032-6.

## Background

### Overview

Owing to a multitude of social and demographic trends, the outpatient sector faces ongoing and significant healthcare imparities, such as urban–rural gaps, both in primary and specialized care [1]. Existing physician shortages intensify rural challenges, including long distances between providers and patients, waiting times, and fragmented care [2, 3]. The age-related and morbidity-related additional demand for healthcare services stands in sharp contrast to the corresponding staffing levels. Physicians are highly challenged, because the intensity and rapidity of demand exceeds their knowledge, skills, and working time, making it impractical to provide the best and adequate care to every patient at every locality [4].

While politics and governance must attract new talent to strengthen staffing levels, individual practices also need to evolve and shift from locally bound, physician-centered consultations to more collaboration and delegation. Thus, it is important to leverage the potential of digital technologies to overcome paper-based and siloed communication [5] as well as geographical distances, and to mitigate regional structural weaknesses [4]. The provision of healthcare services such as diagnosis, education, and treatment across geographic distances via information technologies (ITs) is defined as telemedicine [6]. Telemedicine enables healthcare providers to enhance and extend care delivery beyond healthcare facilities, reducing costs and capturing new value for patients and physicians [7]. Telephone-based and video-based communication approaches are receiving increasing attention, as dramatically demonstrated in the wake of the COVID-19 pandemic and its associated healthcare challenges. This pandemic has placed a massive burden on the healthcare system and has served as a catalyst to foreground the importance of telemedicine approaches for a functioning healthcare system. Telemedical services can aim at, for instance, diagnostics, treatment, patient monitoring, or consultations, and can be applied between patients and providers or between different providers to support clinical decisions [8, 9]. We refer to telemedicine as the telemedical application of online consultations between patients and providers through a digital platform and synchronous video communication via tablets, in line with the research project setting Gesundheitsversorgung 4.0, funded by the Bavarian Ministry of Health.

It is crucial to holistically analyze the factors that support and constrain physicians' acceptance of and societal demand for telemedical technologies. Research into user acceptance of information systems (ISs) has received extensive attention in the past [10–12]. However, IS researchers strongly emphasize the importance of showing how previously validated acceptance models work differently in new contexts [13], including healthcare technologies [14–16]. In literature and practice, there have been various insights into the acceptance and uses of telemedicine [17], specifically online consultations [8, 18–21]. Several studies map influencing factors as well as barriers to acceptance but, e.g., focus on practical implementation efforts or take on the patients’ perspective [22, 23]. Providers’ acceptance strongly impacts development, future implementation, and de facto uses, and therefore patient outcomes [24]. Given that physicians occupy a dual role as providers of medical services while they are users of telemedicine themselves [25], we seek to provide a fundamental model to understand the main factors from the perspective of physicians. Garavand et al. [26] provide the most comprehensive overviews of physician acceptance studies on telemedicine in its variety of applications to date. Thereby, they summarize established core constructs of behavioral models (e.g., self-efficacy, perceived usefulness, perceived ease of use, facilitating conditions, compatibility), organizational and technical factors (e.g., IT equipment, security), socio-oeconomic (e.g., government policies, reimbursement) and cultural factors (e.g., health culture and religious beliefs) as influential for the acceptance of telemedical technologies. Their research highlights a gap as, first, most of the included studies do not differentiate the specific type of telemedicine and look at it generally, and second, as the included studies on telemedical online consultations are set in developing countries and thus vary in the scope of research. We seek to close this research gap and include the physicians’ perspective by asking:

*What are the supporting and constraining factors that influence physicians’ intention to use the telemedical application of online consultations?*

To answer this question, we develop and validate an acceptance model from the perspective of physicians – a key user group in the diffusion process, according to Rogers [24]. Our research model’s theoretical foundation relies on the Unified Theory of Acceptance and Use of Technology (UTAUT), as posited by Venkatesh et al. [12], forming a powerful basis to explain intention to use [13]. Since most previous acceptance models – including UTAUT – aim to predict technology acceptance from a positive utility perspective [27], we adapt the nomological structure of UTAUT to the unique context of telemedicine and integrate potential barriers to and drivers of physicians’ intention to use telemedical technologies. Situating the study in the German healthcare setting provides a homogenous research field with unchanging contextual factors and the resources of a developed country. This consistency makes it possible to focus on the affective and cognitive components of physicians’ intention, examining them as individuals [28]. Thus, we target theoretical insights on explaining acceptance among physicians and practical implications regarding the dissemination of telemedical online consultations.

The remainder of this paper is structured as follows: In the following section, we explain the research’s theoretical background. We then review the literature regarding the acceptance of IS technologies and extrapolate our hypotheses. The succeeding two sections address the research methodology and present our results. To validate the proposed research model, we used partial least squares (PLS) structural equation modeling (SEM), as recommended by Henseler et al. [29] in settings with a high research model complexity in relation to the number of observations (*n* = 127 physicians). Finally, we discuss the research findings, derive theoretical and practical implications, outline limitations, and identify future research avenues.

### The unified theory of acceptance and use of technology

To investigate the acceptance of IS applications such as online consultations, Venkatesh et al. [12] proposed UTAUT, based on a comprehensive literature review and by combining eight established and previously validated research models of technology acceptance [30]. UTAUT merges Theory of Reasoned Action [31], Social Cognitive Theory [32], the Technology Acceptance Model (TAM) [10], Theory of Planned Behavior [33], the Model of PC Utilization [34], the Motivational Model [35], Innovation Diffusion Theory [24], and the C-TAM-TPB Research Model [36]. According to these theories, Venkatesh et al. [12] identified four main antecedents – performance expectancy, effort expectancy, social influence, and facilitating conditions – that influence intention to use and actual usage of IS technologies. Performance expectancy is “the degree to which an individual believes that using the system will help him or her to attain gains in job performance” ([12], p. 447). In the context of telemedicine, we understand this concept as the degree to which physicians believe that telemedicine will help to provide patients with better healthcare services. A second key aspect of UTAUT is effort expectancy, originally referring to the “degree of ease associated with the use of the system” ([12], p. 450). In the telemedicine context, effort expectancy evaluates the perceived required effort of becoming skillful with telemedical technology. As a third variable, Venkatesh et al. ([12], p. 451) proposed social influence – “the degree to which an individual perceives that important others believe he or she should use the new system.” Social influence refers to perceived rules of conduct that are built through compliance, internalization, and identification mechanisms and that are shared by a reference group [37] – here, the physicians themselves, their associations, and the medical research community. As a fourth variable, Venkatesh et al. [12] identified facilitating conditions to potentially predict the intention to use technology. Facilitating conditions are “the degree to which an individual believes that an organizational or technical infrastructure exists to support the use of the system” in a mandatory setting, evaluating the organizational or technical support measures when using a new system ([12], p. 451). However, the research has identified that this effect is fully mediated by effort expectancy [37], indicating no direct relationship between facilitating conditions and intention to use. Further, age, gender, experience, and voluntariness of use were identified as key moderators.

The UTAUT model outperforms each of the eight original models by explaining nearly 70% of the variance of intention to use IS [12, 38]. By including the variables performance expectancy, effort expectancy, and social influence in our model, we rely on UTAUT’s nomological structure. While Venkatesh et al. [12] proposed the construct of facilitating conditions to directly influence intention to use, we did not integrate the concept into our model, since our research context does not involve a mandatory setting. Regarding the relationship between performance expectancy and intention to use, several UTAUT-related theories suggest a direct positive effect. Bandura’s [39] Self-Efficacy Theory proposes that expected outcomes – for instance, attitude or behavioral intention – depend on individuals’ judgments of how well IS systems such as telemedical online consultations will perform in a particular situation. This view is consistent with Social Cognition Theory, indicating that people are more likely to perform a certain behavior if they expect valuable outcomes [32]. Venkatesh et al. [12] identified that performance expectancy showed the strongest influence on intention to use IS among all other observed variables of UTAUT. In the healthcare context, technologies have proven to be useful support tools for physicians, resulting in an increase in the care quality and the efficiency [40]. We therefore hypothesize:

- *H1: Performance expectancy has a direct positive effect on the behavioral intention to use telemedicine.*

According to Self-Efficacy Theory, people particularly avoid behavior “that they believe will exceed their coping capabilities” (40, p. 123). Thus, people tend to perform activities they perceive as easy to cope with [39], resulting in a direct positive effect of effort expectancy on intention to use. Previous acceptance theories posited a direct positive effect, such as TAM [10] or Theory of Planned Behavior [33]. Physicians have low willingness to integrate cumbersome technologies with little time-saving potential into their tight schedules [41]. Thus, if learning and using telemedical online consultations applications is fairly easy, their attitude toward the innovation is more positive [42]. Therefore:

- *H2: Effort expectancy has a direct positive effect on the behavioral intention to use telemedicine.*

Social influence refers to perceived rules of conduct that are built through compliance, internalization, and identification mechanisms and that are shared by a reference group [37]. According to Kelman [43], the compliance and identification mechanism directly affects the intention to use. The positive direct effect can be explained by perceived social pressure (compliance), such as the increasing digitalization of healthcare or increasing competition among physicians, or physicians’ individual affiliation motivations (identification) to an innovative and digital health system. Thus, the “socially expected mode of conduct” [33] induces behavioral intentions, such as the intention to use telemedical online consultations. Therefore:

- *H3: Social influence has a direct positive effect on the intention to use telemedicine.*

Despite the model’s general validity, Venkatesh et al. [13] called for a contextual integration of UTAUT and emphasized the importance of examining the model’s applicability in new surroundings [12, 13]. We therefore adjusted our model and included potential drivers (structural conditions regarding data security, compatibility) as well as barriers (IT anxiety) of acceptance in the telemedicine context.

### Conceptual definitions of drivers of and barriers to conducting online consultations

#### Compatibility

According to Roger’s Innovation Diffusion Theory, compatibility – as the”degree to which an innovation is perceived as being consistent with the existing values, past experiences, and needs of potential adopters” [24, p. 199] – is a key predictor of the acceptance of innovations. Particularly in healthcare, compatibility is likely to affect providers’ intention to use technological innovation owing to a high fragmentation of IT systems, which results in inefficiencies and negative effects on the treatment quality [44]. Schlegel [45] emphasized IT compatibility and the lack of standardized interfaces for authorization, signing, and encryption, which are associated with additional effort and result in hesitant acceptance of digital applications in healthcare. Further, research has already posited the importance of compatibility and effortless integration of technologies in several telemedical contexts, for instance from the patient perspective [46, 47]. When telemedical platforms for online consultations are proposed for use, it is important to consider the need for standardization (e.g., Systematized Nomenclature of Medicine Clinical Terms / SNOMED-CT for consistent semantic and syntactic integration) and interoperability (e.g., Fast Healthcare Interoperability Resources / FHIR for data structure and interfaces) [48, 49].

We refer to an IS system’s compatibility with physicians’ work practices and processes, which is a key factor in telemedicine acceptance [50–52]. Since medical treatment involves various tests and information, and medical diagnosis and healthcare provision mistakes should be avoided, the compatibility of different systems has a key role [50]. If telemedical online consultations are not perceived to be compatible with existing work practices and processes, they may not be perceived as easy to use and may therefore not be accepted by physicians. It is likely that systems cannot be perceived as easy to use if they are not compatible with established processes [53]. Therefore:

- *H4: Compatibility has a direct positive effect on the intention to use telemedicine*

#### IT anxiety

The usage of novel technology interferes with traditional working practices and changes routines [11], in some cases causing uncertainty and the perception of a negative attitude toward IT [54, 55]. This so-called IT anxiety is defined as the “negative affective reaction toward computer use” (56, p. 349). In contrast to Venkatesh et al. [12], who did not include anxiety in UTAUT, we argue that there is a reason why IT anxiety lowers the intention to use new ITs in a healthcare context. Based on two theoretical underpinnings, we propose that IT anxiety influences the intention to use telemedicine; however, this is explained through performance and effort expectancy. First, we refer to classic theory on anxiety research, showing that anxiety negatively influences cognitive responses and particularly process expectancy [56], such as the expected effort or performance. This assumption was strengthened by Morris et al. [57], identifying that the cognitive component of anxiety impacts on expectancies. Classic theory of anxiety is consistent with the second rationale for a direct relationship between IT anxiety and expectancies, derived from Bandura’s [32] Social Cognitive Theory. The theory proposes that anxiety impacts on expectancies and vice versa. Thus, higher IT anxiety levels lead to higher expected effort levels regarding the use of telemedical online consultations.

Adapted to the health context, Riepe and Schwanenflügel [58] posited that a trusting relationship between doctors and patients is the basis of successful healthcare and a prerequisite for optimal diagnosis and therapy. Personal physician–patient contact seeks to ensure that physicians generally obtain a specific and comprehensive assessment of the patient’s state of health, rather than relying solely on patient descriptions or information provided by third parties. This gains importance when considering that certain clinical pictures can only be identified through external symptoms, which would remain undetected by the mere transmission of a condition's description without physical examination [59]. Thus, and in line with previous research [47, 60, 61], physicians are likely to refuse accepting IT applications in order to prevent a damaging impairment of the trust relationship owing to the physical distance [62] or to avoid medical misjudgment caused by using telemedicine [59], such as online consultations. Therefore:

- *H5: IT anxiety has a direct negative effect on the performance expectancy of telemedicine.*
- H5*: The relationship between IT anxiety and the intention to use telemedicine is mediated by performance expectancy.
- *H6: IT anxiety has a direct negative effect on the effort expectancy of telemedicine.*
- H6*: The relationship between IT anxiety and the intention to use telemedicine is mediated by effort expectancy.

#### Structural conditions regarding data security

The variety of data and cybersecurity issues associated with telemedicine services requires comprehensive security regulations and policies to maintain and manage appropriate measures in telemedicine environments [63] in which physicians engage. Former research has identified perceived data security as a key predictor among physicians in the implementation process and their intention to use an e-health application [64]. Thus, as a second antecedent, we integrated the variable structural conditions regarding data security, evaluating a privacy-related component of the technical infrastructure. According to Vroom’s [65] Expectancy Theory, individuals seek to minimize negative consequences as a result of their behavior. Despite the risk of undesirable outcomes of technology use – such as the loss of patient data – telemedical technology is perceived by physicians to be more secure than traditional approaches of data storage, given to a strict regulatory, technical, and ethical framework on governmental regulations [66]. Thus, we expect physicians’ perception of national and organizational security practices in terms of medical data to be a key antecedent to explain the intention to use online consultations [16, 27].

- *H7: The importance of structural conditions regarding data security has a direct positive effect on the performance expectancy.*
- H7*: The relationship between the importance of structural conditions regarding data security and the intention to use telemedicine is mediated by performance expectancy.
- *H8: The importance of structural conditions regarding data security has a direct positive effect on the effort expectancy.*
- H8*: The relationship between the importance of structural conditions regarding data security and the intention to use telemedicine is mediated by effort expectancy.

**Fig. 1:**
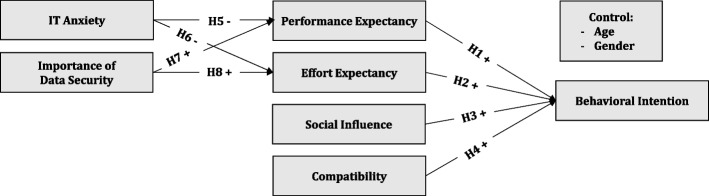
Proposed Research Model

Figure 1 summarizes up the hypotheses and the proposed research model. It further encompasses the common demographic control variables age and gender.

## Methods

### Research context: online consultations

To validate our model and evaluate the intention to use online consultations, we distributed an online questionnaire among German physicians in the context of the research project Gesundheitsversorgung 4.0. This project accelerates the treatment of patients via a direct and secure video and audio connection with simultaneous access of general physicians to the corresponding digital patient file for a secure exchange of patient-related data. On the patient side, the default application is the tablet. Utilizing a digital platform linked to the electronic care record, the patient or caregiver can input the patient's health status and securely transmit it to the overseeing general physician who receives the message either on their office computer or tablet device through a push notification. Messages are categorized by color, ranging from "white" for non-urgent matters to "red" for urgent actions, such as immediate contact with the patient, preferably on the same day. Subsequently, the primary care physician can respond, seek additional details, or schedule online consultations based on the message's priority within the application. These functionalities ensure continuous interaction and communication as well as fast responses to occurring medical events but are not designed to replace emergency medicine. Thus, online consultations are an application of telemedicine technologies and seek to establish better-quality medical care and efficiency. Furthermore, they are intended for use primarily in nursing facilities, such as old people’s homes, nursing homes, and medical care.

### Operationalization of the constructs

Previously validated scales served as a starting point to build our measurement model. Since our model relies on UTAUT, we took items from Venkatesh et al. [12] that measure performance expectancy, effort expectancy, and intention to use. For the variable social influence, we used Ajzen’s [33] scale. To assess the importance of structural conditions regarding data security, we used Dünnebeil et al.’s [16] items. Regarding the antecedents, we chose Moore and Benbasat’s [67] scale for compatibility and Venkatesh’s [55] scale for IT anxiety for the telemedicine context. However, we had to translate the items from English into German due to the German research context.

We conducted two rounds of card-sorting, assessing the validity and reliability of our measurement model following the procedure proposed by Wood and Wood [68] and Moore and Benbasat 67). Thereby, we adjusted the raw items iteratively regarding wording, language, and formality to account for the respective research context and to provide a common style. The procedure was as follows: first, the participants were given written instructions on how to complete the card sorting procedure. Next, the participants familiarized themselves with the context of the research project. For this purpose, a short project description and an illustration of the research model were provided. In addition, we provided definitions of the model’s constructs to achieve a consistent understanding among the participants. Then, the participants assigned the items – presented in a randomized order – to the construct that, in their opinion, fits the description best. If participants did not identify a fit between item and construct, they selected the option “unclear”. Additionally, participants had the opportunity to provide qualitative comments on the items in a given space next to the items. In the first round, 82% (14 of 17) of the participants concluded the card sorting. We achieved a relatively low hit ratio of 61%, while the lowest score was 44% and the highest score was 90%. The qualitative feedback hinted toward some translation issues. Based on the feedback and a further literature review, we revised the wording of the constructs’ definitions and the items. After that, we conducted a second round of card sorting. 100% (6 of 6) of the (new) participants concluded the second round of card sorting. With an overall hit ratio of 83%, the measurement model seems satisfying, as does the range of the lowest score (79%) to the highest score (90%). Consequently, we elicited items with a hit ratio lower than 80% and integrated the remaining items included in the second card sorting procedure in the final questionnaire (Table 1).

**Table 1:** Summary of the questionnaire

Construct	Items	
Intention to use (IU) [12]	IU1	I intend to use online consultations in the future
	IU2	I plan to use online consultations in the future
Performance expectancy (PE) [12]	PE1	Using online consultations could increase healthcare for patients in old people’s homes and nursing homes
	PE2	Online consultations would be a useful extension of existing treatment methods
Effort expectancy (EE) [12]	EE1	I expect online consultations to be easy to understand and use
	EE2	I expect to find online consultations easy to use
Social influence (SI) [33]	SI1	I think my colleagues would support the use of online consultations
	SI2	I think our patients would support the use of online consultations
The importance of structural conditions regarding data security (DS) [16]	DS1	National security standards for the handling of patients’ medical data are necessary
	DS2	Committing standards for the handling of patients’ medical data are necessary for my practice
	DS3	It’s important to me to be able to extensively inform my patients about the use of their medical data
Compatibility (CO) [67]	CO1	Using online consultations is compatible with the way I want to work with patient data
	CO2	I think that using online consultations fits well with the way I like to interact with my colleagues
	CO3	Using online consultations fits into my work style
IT anxiety (IA) [55]	IA1	Working with a tablet makes me nervous
	IA2	I feel threatened when others talk about tablets
	IA3	Tablets make me feel uncomfortable
	IA4	I get a sinking feeling when I think of trying to use a tablet
	IA5	Tablets make me feel uneasy

### Data collection

Data collection took place from December 2018 to February 2019. We distributed a web-based questionnaire via institutional e-mail addresses among 300 German physicians. To ensure an appropriate understanding among the participants, a detailed description regarding the relevant aspects of online consultations was included in the questionnaire design (see Additional File 1). Thereby, we emphasized that we were interested in physicians’ perspectives as potential users and refrained from integrating contextual components (e.g., reimbursement regulations) that would address more organizational perspectives. Participation in the study was entirely voluntary without providing any incentives. For the questionnaire guide, please see Additional File 2.

## Results

### Demographics of the sample

We received 127 completed questionnaires (response rate: 42.33%). The sample demographics are illustrated in Table 2. The mean age was 42,24 years, the gender ratio split in 35.4% female and 60.6% male (3.9% did not want to disclose this information). The specification showed a large proportion of general medicine physicians (83.5%) and a comparatively lower proportion (16.5%) of medical specialists, e.g., geriatricians, internists, and oncologists.

**Table 2:** Demographics of the Sample

Demographics	Descriptive statistics (*n* = 127)	
Age		Mean 42.24, S.D. 8.44; range 23 to 57
	20 to 30	07.1% (*n* = 9)
	31 to 40	30.7% (*n* = 39)
	41 to 50	45.7% (*n* = 58)
	51 to 60	12.6% (*n* = 16)
	Prefer not to say	03.9% (*n* = 5)
Gender	Female	35.4% (*n* = 45)
	Male	60.6% (*n* = 77)
	Prefer not to say	03.9% (*n* = 5)
Specification	General medicine	83.5% (*n* = 106)
	Medical specialist	16.5% (*n* = 21)

### Measurement model

We embedded the factors that determine the intention to use telemedicine technologies in a theoretically developed cause-effect relationship model. Since our model has a high complexity compared to the number of observations (*n* = 127 physicians), we used PLS-SEM to validate the research model. We used the statistical software SmartPLS 3 to estimate our model’s parameters. As recommended by Hair et al. [69], we used path weighting, a maximum of 300 iterations, and a stop criterion of 10^–7^ in the PLS-SEM algorithm settings. The constructs in our model represent latent variables. Each latent variable requires a set of observable indicators for a reliable and valid measurement procedure. Although we used previously validated scales as indicators of our variables, it is necessary to demonstrate the measurement model’s reliability and validity to ensure meaningful results from the structural model. Thus, we followed the recommended statistical procedures to ensure our results’ salience.

Convergent validity was assessed based on factor loadings, composite reliability, and average variance extracted (AVE). Factor loadings should be over 0.5 [70], composite reliabilities over 0.8 [71], and the minimum for the AVE is 0.5 [70]. Further, we analyzed Cronbach’s $\alpha$, which is commonly used to test for the internal consistency of the variables. As illustrated in Table 3, all estimated indices were above the recommended thresholds, except the value of Cronbach’s $\alpha$ of the variable social influence. However, since all other indices were satisfactory, we kept the variable in the model.

**Table 3:** Internal Reliability and Convergent Validity of the Measurements

		Internal reliability	Convergent and discriminant validity		
Construct	Item	Cronbach’s α	Factor loading	Composite reliability	AVE
Intention to use telemedicine (IU)	IU1	.88	.94	.93	.81
	IU2		.95		
	IU3		.80		
Performance expectancy (PE)	PE1	.89	.95	.95	.90
	PE2		.96		
Effort expectancy (EE)	EE1	.83	.93	.92	.86
	EE2		.92		
Social influence (SI)	SI1	.69	.87	.87	.77
	SI2		.88		
The importance of structural conditions regarding data security (DS)	DS1	.72	.84	.84	.64
	DS2		.85		
	DS3		.71		
IT anxiety (IA)	IA1	.84	.76	.89	.66
	IA2		.85		
	IA3		.81		
	IA4		.83		
Compatibility (CO)	CO1	.88	.93	.92	.80
	CO2		.89		
	CO3		.96		

Discriminant validity evaluates the degree to which measures of different variables are distinct [72], and is established by showing that the square roots of the AVEs are greater than the corresponding off-diagonal inter-construct correlations [70, 73], as illustrated in Table 4. We also consulted Henseler et al.’s [73] heterotrait-monotrait (HTMT) criterion as additional measurement to determine discriminant validity. As shown in Table 5, all the HTMT values were below 0.85, representing discriminant validity in all constructs [73].

**Table 4:** Inter-Construct Correlations and Square Roots of AVE

Construct	IU	PE	EE	SI	DS	IA	CO
IU	**.90**						
PE	.67	**.95**					
EE	.40	.38	**.93**				
SI	.65	.65	.37	**.88**			
DS	.19	.24	.34	.18	**.80**		
IA	-.29	-.37	-.40	-.31	-.14	**.81**	
CO	-.03	.02	.08	.03	.12	.04	**.90**

Elements in bold on the diagonal are square roots of the AVE

**Table 5:** Heterotrait-Monotrait Criterion

Construct	IU	PE	EE	SI	DS	IA	CO
IU							
PE	.76						
EE	.47	.44					
SI	.83	.82	.48				
DS	.23	.28	.43	.24			
IA	.31	.38	.46	.35	.21		
CO	.04	.05	.11	.06	.17	.08	

Owing to the use of a single method, we tested for common method bias. The Harman single-factor test assumes that the presence of common method variance is indicated by the emergence of a single or general factor that accounts for most of the covariance among measures [74]. An exploratory factor analysis without rotation illustrated that six factors were extracted, with the first factor explaining only 25.88% of the variance, which does not account for most of the covariance among measures.

### Structural model and testing of the hypotheses

After ensuring that our measurement model is valid, we used the PLS algorithm with 5,000 bootstraps to analyze the proposed hypotheses’ salience. A total of 55% (R^2^ = 0.55) variation of the main dependent variable intention to use was explained by the exogenous variables in the research model. We tested the proposed hypotheses by analyzing the standardized path coefficients between constructs together with the corresponding *P*-values calculated by bootstrapping procedure (5,000 samples). The intention to use is mostly determined by performance expectancy, which positively affected physicians’ intention to use telemedicine (H1, β = 0.397, *P* < 0.001). As indicated, effort expectancy (H2, β = 0.134, *P* = 0.03) and social influence (H3, β = 0.337, *P* < 0.001) significantly influenced the intention to use. Surprisingly, we found no direct relationship between compatibility and the intention to use (H4, β = -0.067, *P* = 0.325). Regarding the antecedents of performance and effort expectancy, we could confirm that IT anxiety to negatively affect both performance (H5, β = –0.342, *P* < 0.001) and effort expectancy (H6, β = –0.364, *P* < 0.001). The analysis also revealed significant outcomes regarding the relationship between the importance of data security and performance (H7, β = 0.193, *P* < 0.001) and effort expectancy (H8, β = 0.295, *P* < 0.001). The results were controlled for age (β = 0.005, *P* = 0.934) and gender (β = 0.064, *P* = 0.320.) differences.

**Fig. 2:**
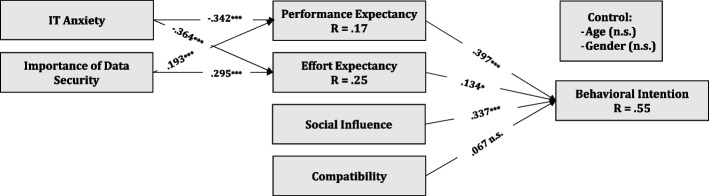
Research Model with Standardized Regression Weights Notes: (n.s. = not significant; * *P* < .05; ** *P* < .01; *** *P* < .001)

Figure 2 summarizes the results.

Further, we followed Zhao et al. [75] and estimated an extended PLS model [76] with the relevant direct path between IT anxiety and behavioral intention to test for mediation. We observed no significant differences in the inner and outer estimates compared to the initial model and thus conducted another bootstrapping analysis (5,000 samples). According to our analysis, performance expectancy fully mediated IT anxiety’s effect on the behavioral intention (β^direct^ = 0.022, *P* = 0.713; β^indirect^ = -0.138, *P* < 0.001), while effort expectancy did not (β^direct^ = 0.022, *P* = 0.713; β^indirect^ = -0.051, *P* = 0.086). Regarding the importance of data security, performance expectancy fully mediated IT anxiety’s effect on the behavioral intention (β^direct^ = -0.002, *P* = 0.978; β^indirect^ = -0.196, *P* < 0.001), as did effort expectancy (β^direct^ = -0.002, *P* = 0.978; β^indirect^ = -0.297, *P* < 0.001).

## Discussion

### Theoretical contribution

From a theoretical perspective, we built on previous acceptance research in healthcare (e.g., [8, 18–21]) and further empirically examined physicians’ intention to conduct telemedical online consultations. We identified drivers and barriers that explain behavioral intention. Most remarkably, we identified two antecedents – IT anxiety and the importance of data security – that significantly determine the intention to use online consultations (indirectly). By relying on UTAUT as the underlying foundation of our research model [12], we have broadened UTAUT’s nomological structure and provided a theoretical rationale for which factors influence the intention to conduct online consultations. In line with UTAUT [12], Innovation Diffusion Theory [24], and the TAM [10], performance expectancy significantly predicted physicians’ intention to use, affirming existing healthcare technology acceptance research [77]. Further, effort expectancy significantly predicted intention to use. Thus, physicians are likely able to assimilate novel technologies if the expected effort is manageable. Since social influence correlated with the intention to use telemedicine among physicians, we interpret the results as physicians being highly dependent on subjective norms and their colleagues’ behavior when intending to use telemedicine. In line with recent research that calculated significant influencing effects of physicians as early adopters on others’ implementation behavior [78], we emphasize the importance of social influence, for instance, colleagues who act as role models for other physicians when implementing digital technologies such as telemedical online consultation. Thus, questions arise about how to build and exploit the potential of social connections and groups among physicians in the heterogeneous healthcare landscape (e.g., independent solo practitioners, healthcare alliances, hospitals). Somewhat surprisingly, compatibility did not relate significantly to behavioral intention, despite research claims that a high fragmentation of IT systems usually leads to inefficiencies and negative effects on the treatment quality [44]. Further, the research states that the technical ability and affinity to work with telemedicine impacts on compatibility on an individual level [53]. Our unexpected result could be caused by a general affinity with technological innovation or insufficient experience on the part of the participating physicians with telemedical online consultations. According to our model, IT anxiety is negatively associated with performance expectancy (direct), effort expectancy (direct), and intention to use (indirect, mediated via performance expectancy). This is in line with former research that posited IT anxiety’s role as a key inhibitor of technology acceptance in organizations generally [13] and in e-health contexts from a patient’s perspective [47]. Here, physicians who are anxious about telemedical online consultations associate it with lower performance and higher effort. In other words, it is not the expectancy per se that hinders them from conducting online consultations, but the underlying anxiety, which acts like a fog in the evaluation of expectancies. While Aggelidis et al. [61] as well as Tsai et al. [47] observed these relationships among patients and healthcare workers generally, we turned to physicians, providing an important perspective that extends previous studies. Here, possible rationales can be that, regarding novel technologies, physicians fear that telemedicine may negatively affect traditional working practices and may change routines, impairing performance and amplifying the fear that the new technology would unpleasantly increase the needed effort to interact with patients. These phenomena were also observed during the accelerated transition to telemedicine due to COVID-19 [60]. Solutions for healthcare organizations, IT providers, and other relevant stakeholders to overcome anxiety-related obstacles may be to develop familiarity with the technology [79], for instance, through extensive user training, and to enhance collaboration and knowledge sharing, e.g., by asking tech-savvy colleagues and students for help [60]. In addition to IT anxiety, the importance of structural conditions regarding data security influenced performance expectancy (direct), effort expectancy (direct), and intention to use (indirectly) – as we had theorized. According to Jalali et al. [80], security concerns about telemedicine mainly focus on privacy and data protection (patient information safety), owing to threats of malicious hacking or the accidental disclosure of sensitive information. Considering the fundamental values of medical professionals [81], as well as existing research into the protection and confidentiality of patient-related data [82], physicians constantly work with sensitive data in their daily work and are aware of the importance of complying with data protection guidelines and informed patient consent. When national regulations and standards are sufficiently enforced (e.g., in software), physicians can have confidence that all stakeholders act within a regulated and safer environment. We argue that trust in regulation allows physicians to act more confidently while handling telemedical applications, which in turn increases the intention to use the technologies.

### Practical contribution

From a practical perspective, this work provides important access to the implementation of online consultations for stakeholders in the physician–patient relationship (e.g., physicians, patients, physician associations, education programs, technology providers). First, it will be crucial to demonstrate telemedicine’s ability to fulfill the needs of physicians, who tend to accept these technologies only when the underlying value or the expected performance for patients and their individual practices materialize. Further, our results emphasize the importance of social influence, leading to implications for stakeholders such as physician associations and their role as a key link in and platform for exchange between physicians. Organizations, and associations that seek to implement online consultations should educate multipliers as a practical lever to promote the use of online consultations. Thus, a more active role is necessary – as Han et al. [83] posited that social influence’s effect is especially high among physicians with little experience regarding the application. Establishing regular community meetings and networking can support the continual exchange and convergence of physicians as a group [84]. Despite the increasing use of ITs in the workplace and personal life, our results demonstrated IT anxiety’s negative effect on performance and effort expectancy. The provision of proper user training and demonstrations is vital for directing and solidifying physicians’ perceptions of telemedicine’s expected performance [85–87]. Communicating a system’s operations and usefulness for physicians and patients may open “black boxes’”, ultimately leading to higher intention to use online consultations [88]. Further, the adoption of telemedicine could be enhanced with more intense involvement of providers in the IT design (to promote user-centricity) and by facilitating other requirements, such as adequate reimbursement and administrative support [89, 90]. Our results demonstrated the importance of structural conditions regarding data security. Accordingly, politicians and regulating institutions are called on to provide the framework conditions to allow for the introduction of telemedical applications. For an overview of the key insights from a theoretical and practical point of view and derived recommendations, see Table 6.

**Table 6:** Key Insights and Derived Recommendations

Construct	Key Insights	Derived Recommendations
SI	• Colleagues can serve as role models and foster acceptance of online consultations • Governing organizations such as physician associations act as key link for exchange between physicians regarding opinions on online consultations	• Social connections and groups (meetings and networking) among physicians should be built and nurtured • Education and establishment of multipliers allows them to explain the use of telemedical online consultations to reach convergence
IA	• Fear of change in working practices, impaired performance, increased effort for patient interaction can feed IT anxiety towards online consultations	• Development of familiarity through user training and demonstrations, collaboration, knowledge sharing, involvement in IT design and facilitating monetary incentives or administrative requirements can reduce IT anxiety
DS	• Physicians have awareness of sensitive patient data and reflect the importance of online consultations’ compliance with data protection guidelines	• Enforcement of national regulations, standards, and low-entry concepts for the implementation of framework conditions builds confidence in adherence of telemedical online consultations

### Limitations and future research opportunities

Owing to the nature of our research, this study has limitations, which offer opportunities for future research. First, with a sample size of 127 participants and a focus on German physicians, we suggest replicating the study in a broader context. Our physicians were, on average, 42.24 years old, significantly lower than the German average of 53.67 years. Further, the share of male participants was proportionally larger than the share of female participants. Thus, a broader replication of the study to revalidate our investigations’ results is needed. Second, since our cross-sectional study design offered only data at a single point in time, a longitudinal examination of the diffusion of online consultations may contribute valuable details to physicians’ acceptance of telemedicine and thereby tackle the challenges of regional health imparities.

Third, although the research has shown that intentions are a good predictor of de facto behavior [10, 12], intentional and de facto evaluations of acceptance factors of technologies could be different [91]. Thus, concerning the future acceptance of telemedicine, researchers could investigate de facto behavior instead of intention to use. Fourth, compatibility’s nonsignificant effect on intention to use is very interesting yet lacks appropriate reasoning. One explanation may be that, owing to the project setting, our participants were physicians with an affinity for technological innovation. While we did control for age and gender, future research should shed further light on compatibility’s role in the intention to use the technology. Finally, investigating which factors influence IT anxiety and structural conditions regarding data security is a promising starting point for an improved understanding of structural requirements in telemedical acceptance processes.

As the present study focuses on physicians as individuals, further studies should investigate the context in which telemedicine applications are used. This will allow for more practice-oriented results—highlighting barriers or facilitators to the use of telemedicine that arise from everyday medical practice. These include, e.g., financial aspects of using telemedicine, such as reimbursement for delivered services, but also the availability of investment budgets that would help physicians drive technological change [92, 93]. E.g., the German Ministry of Health passed an e-health law that explicitly allowed reimbursement for telemedicine services just as late as 2015 and promoted telehealth in 2019 with the Digital Healthcare Act [94]. Moreover, future studies should include legal regulations and the existence of certain structures, for example, regarding the approval of physicians and software for telemedical treatment, but also of treatment as an adequate part of standard care. Additionally, clear regulations on liability and responsibilities must be set so that both physicians and patients feel confident [95]. In Germany, e.g., solely the National Association of Statutory Health Insurance (a self-governing body for assessment and distribution of medical suppliers) certifies telemedicine providers [96]. Furthermore, a deeper reflection of the available IT infrastructure and network connectivity, a basic prerequisite for telemedical care, is necessary [92]. According to the German Federal Ministry of Transport and Digital Infrastructure, some rural regions still lack access to a private broadband connection [96]. This condition may dominate lower-income countries or may be irrelevant in highly digitized countries, e.g., Estonia [97]. Subsequent processing of this knowledge in a cross-national approach could provide further insights into the structural requirements of telemedical acceptance processes. In the wake of the overall systemic burden of the COVID-19 pandemic, increased use of telemedicine is being recorded across various healthcare sectors and disciplines [98–101]. Physicians and patients gain more experience with telemedicine as they are forced to use technologies to reduce unnecessary physical contact and minimize potential infectious exposures [102]. Thus, comparing the use of telemedicine and factors of physicians’ acceptance before, during, and post-COVID-19 is a key future research avenue because the underlying behavioral and psychological constructs are impacted. As this study is set before COVID-19, a comparison of all stages will allow for a better understanding of the drivers of telemedicine, with the aim to scale up substantially so that its increased use is not just a quick emergency fix for the pandemic but continues to spread in a sustainable way after the crisis is over [103]. For instance, compatibility with existing work practices may evolve in line with how work culture and habits evolve [104]. If stakeholders in medical processes (e.g., collaborating physicians and patients) unanimously use telemedicine for specific tasks, they could create a smooth working environment for one another and could increase compatibility. This may encourage physicians to perceive telemedicine as the most efficient, default mode of interaction for online consultations. IT anxiety may decrease overall as the exposure to technology progresses in workplaces and personal life helps individuals feel more comfortable interacting with smartphones, tablets, computers, and various communication platforms, such as video-based communication (e.g., via Zoom, WebEx, or Microsoft Teams) [25]. One consideration that becomes important when contextual factors change dramatically, as they did in the pandemic, is the weighting between the influence of individual physicians’ antecedents like IT anxiety and medical needs. Physicians have very strong moral and professional obligations [105] and might put the interest of treatment and aid above their individual fear of using IT. In a pandemic environment, where medical service delivery is limited by isolation obligations of both patients and providers, this trade-off could be in favor of service delivery, even if IT anxiety remains as a result. However, regarding the importance of data security, Jalali et al. [80] reported an increased risk of cyberattacks on the healthcare sector during the pandemic; this may again increase IT anxiety. The protection of telemedicine platforms is complex and requires a multidisciplinary approach. Physicians’ awareness of data security may be influenced by their education and staff training, for instance, in simulated cyberattacks that make physicians more familiar with the issue and with safety measures [80].

## Conclusions

Increasing demand for healthcare confronts outpatient physicians with the challenge of providing medicine of consistent quality, regardless of reduced capacity. In this context, telemedical approaches such as online consultations have proven to be successful in boosting efficiency and closing regional gaps. In any consideration of technological innovation in healthcare, one must analyze physicians’ acceptance. 

We have examined the constraining and supporting factors that influence physicians’ intention to use telemedical online consultations. Our model appropriately explains physicians’ behavioral intention to use online consultations, underlining UTAUT's applicability in healthcare contexts. By expanding UTAUT’s nomological structure, we identified IT anxiety as a key constraining factor, raising the question of how to appropriately lower concerns in physicians – both theoretically and practically. 

While technological developments are emerging rapidly – especially in healthcare – we are still in the early stages of adoption in and integration into physicians’ working routines. Thus, regulators, associations, physicians, and patients are being pressured to enhance data protection-compliant technological integration in the physician–patient relationship to offer state-of-the-art treatments for everyone.

### Supplementary Information

**Additional file 1.**

**Additional file 2.**

## Data Availability

The datasets analyzed during the current study are available from the corresponding author on reasonable request.
