# Supplementary material for: Examining supporting and constraining factors of physicians’ acceptance of telemedical online consultations: a survey study
Source: BMC Health Serv Res. 2023 Oct 19;23:1128. doi: 10.1186/s12913-023-10032-6 (PMC10588103; doi:10.1186/s12913-023-10032-6)
Supplement: Supplementary file 1 — Additional file 1. [file 12913_2023_10032_MOESM1_ESM.pdf]

Additional File 1: Information Material for Participants

Dear participants,

Thank you very much for your participation in our recent online survey on the topic of telemedicine utilization in primary care. This theme is part of the "Gesundheitsversorgung 4.0" project by the Bavarian State Ministry of Health and Care. Your valuable input helps us develop a comprehensive understanding of this subject and gain better insights.

We would like to take this opportunity to explain what we mean by "online consultations" in the context of telemedicine. Online consultations involve an innovative method where general practitioners and patients (often with the support of caregivers in the nursing/ geriatric field) interact through digital platforms.

Thanks to the online consultation, the general physician and patient no longer need to be physically present for every medical examination. Based on the digital patient record, a message regarding the health status is created and encrypted, then transmitted to the attending general physician. The general physician receives the message in their office on a computer or tablet. The scale ranges from a non-urgent message marked "white" to a "red" marked message. This color coding allows the physician to prioritize messages and integrate them into their practice workflow accordingly. For instance, a red message indicates that the physician will promptly contact the patient, preferably on the same day, whereas the lowest level might have one to two days' time.

Simultaneously, the physician can securely access and review patient data online within a protected network. After evaluating this data along with their own existing patient records, the physician has several options:

- They can ask for further information, and possibly decide to visit the patient's home for (an adjustment of) treatment.
- If a home visit isn't absolutely necessary but a personal conversation between patient and physician is needed, an online consultation can be scheduled. By utilizing visual and auditory contact with patients and caregivers via video and audio consultation, physicians can conduct progress checks and treatments.

This advanced form of consultation enables us to overcome barriers in terms of availability and distance, enhancing access to medical care. Please take a moment to envision this setup then proceed to answer the questions. Please note that online consultations in this setting are not intended to replace emergency medicine but serve routine purposes!

Your opinions and thoughts are extremely important to us, and we are eager to gain further insights from your participation in the survey. Once again, thank you for your support in our efforts to bridge gaps in regional healthcare provision.

Best regards,

Your Gesundheitsversorgung 4.0-Team
