# Supplementary material for: Examining supporting and constraining factors of physicians’ acceptance of telemedical online consultations: a survey study
Source: BMC Health Serv Res. 2023 Oct 19;23:1128. doi: 10.1186/s12913-023-10032-6 (PMC10588103; doi:10.1186/s12913-023-10032-6)
Supplement: Supplementary file 2 — Additional file 2. [file 12913_2023_10032_MOESM2_ESM.pdf]

Additional File 2: Questionnaire Guide

1. Which professional group do you belong to?

General Practitioner	Specialist

2. If Specialist is selected, please provide your specialty.

3. Please provide your age.

4. Please select your gender.

Female	Male

5. How many years have you been working in your profession?

Please answer the following questions:

6. I intend to use online consultations in the future.

Strongly Disagree	Disagree	Somewhat Disagree	Neutral	Somewhat Agree	Agree	Strongly Agree

7. I plan to use online consultations in the future.

Strongly Disagree	Disagree	Somewhat Disagree	Neutral	Somewhat Agree	Agree	Strongly Agree

8. Using online consultations could increase healthcare for patients in old people's homes and nursing homes.

Strongly Disagree	Disagree	Somewhat Disagree	Neutral	Somewhat Agree	Agree	Strongly Agree

9. Online consultations would be a useful extension of existing treatment methods.

Strongly Disagree	Disagree	Somewhat Disagree	Neutral	Somewhat Agree	Agree	Strongly Agree

10. I expect online consultations to be easy to understand and use.

Strongly Disagree	Disagree	Somewhat Disagree	Neutral	Somewhat Agree	Agree	Strongly Agree

11. I expect to find online consultations easy to use.

Strongly Disagree	Disagree	Somewhat Disagree	Neutral	Somewhat Agree	Agree	Strongly Agree

12. I think my colleagues would support the use of online consultations.

Strongly Disagree	Disagree	Somewhat Disagree	Neutral	Somewhat Agree	Agree	Strongly Agree

13. I think our patients would support the use of online consultations.

Strongly Disagree	Disagree	Somewhat Disagree	Neutral	Somewhat Agree	Agree	Strongly Agree

14. National security standards for the handling of patients' medical data are necessary.

Strongly Disagree	Disagree	Somewhat Disagree	Neutral	Somewhat Agree	Agree	Strongly Agree

15. Committing standards for the handling of patients' medical data are necessary for my practice.

Strongly Disagree	Disagree	Somewhat Disagree	Neutral	Somewhat Agree	Agree	Strongly Agree

16. It's important to me to be able to extensively inform my patients about the use of their medical data.

Strongly Disagree	Disagree	Somewhat Disagree	Neutral	Somewhat Agree	Agree	Strongly Agree

17. Using online consultations is compatible with the way I want to work with patient data.

Strongly Disagree	Disagree	Somewhat Disagree	Neutral	Somewhat Agree	Agree	Strongly Agree

18. I think that using online consultations fits well with the way I like to interact with my colleagues.

Strongly Disagree	Disagree	Somewhat Disagree	Neutral	Somewhat Agree	Agree	Strongly Agree

19. Using online consultations fits into my work style.

Strongly Disagree	Disagree	Somewhat Disagree	Neutral	Somewhat Agree	Agree	Strongly Agree

20. Working with a tablet makes me nervous.

Strongly Disagree	Disagree	Somewhat Disagree	Neutral	Somewhat Agree	Agree	Strongly Agree

21. I feel threatened when others talk about tablets.

Strongly Disagree	Disagree	Somewhat Disagree	Neutral	Somewhat Agree	Agree	Strongly Agree

22. Tablets make me feel uncomfortable.

Strongly Disagree	Disagree	Somewhat Disagree	Neutral	Somewhat Agree	Agree	Strongly Agree

23. I get a sinking feeling when I think of trying to use a tablet.

Strongly Disagree	Disagree	Somewhat Disagree	Neutral	Somewhat Agree	Agree	Strongly Agree

24. Tablets make me feel uneasy.

Strongly Disagree	Disagree	Somewhat Disagree	Neutral	Somewhat Agree	Agree	Strongly Agree
